# Pinpointing change in virtual reality assisted treatment for violent offenders: a pilot study of Virtual Reality Aggression Prevention Training (VRAPT)

**DOI:** 10.3389/fpsyt.2023.1239066

**Published:** 2023-11-16

**Authors:** David Ivarsson, Carl Delfin, Pia Enebrink, Märta Wallinius

**Affiliations:** ^1^Evidence-based Forensic Psychiatry, Department of Clinical Sciences Lund, Faculty of Medicine, Lund University, Lund, Sweden; ^2^Swedish Prison and Probation Service, Norrköping, Sweden; ^3^Centre for Ethics, Law and Mental Health, Department of Psychiatry and Neurochemistry, Institute of Neuroscience and Physiology, Sahlgrenska Academy, University of Gothenburg, Gothenburg, Sweden; ^4^Division of Psychology, Department of Clinical Neuroscience, Karolinska Institutet, Stockholm, Sweden; ^5^Research Department, Regional Forensic Psychiatric Clinic, Växjö, Sweden

**Keywords:** virtual reality, offender treatment, aggression, prison, pilot study, violence

## Abstract

Preventing relapse into violence and its destructive consequences among persistent re-offenders is a primary concern in forensic settings. The Risk-Need-Responsivity framework models the best current practice for offender treatment, focused on building skills and changing pro-criminal cognitions. However, treatment effects are often modest, and the forensic context can obstruct the delivery of interventions. Developing treatments for offenders should focus on the best method of delivery to make “what works work.” Virtual reality (VR)-assisted treatments such as Virtual Reality Aggression Prevention Training (VRAPT) are a new and innovative approach to offender treatment. This pilot study followed 14 male violent offenders who participated in VRAPT in a Swedish prison context and measured changes from pre-treatment to post-treatment and 3-month follow-up in targeted aggression, emotion regulation, and anger. It also investigated potential impact factors (pro-criminal cognitions, externalizing behaviors, psychosocial background, and childhood adverse experiences). In Bayesian linear mixed effects models, participants showed a high probability of change from pre-treatment to post-treatment and to follow-up on all outcome measures. All outcome measures demonstrated a low probability of change from post-treatment to follow-up. Analysis of reliable change showed that participants’ results ranged from recovery to deterioration. We discuss the implications of the study for VRAPT’s impact on the target group, those who might benefit from the approach, and suggested foci for future studies in the field of VR-assisted offender treatment. The study was preregistered at the International Standard Randomized Controlled Trial Number registry (https://doi.org/10.1186/ISRCTN14916410).

## 1. Introduction

Youth and adults cared for and incarcerated in various forensic institutions constitute a heterogeneous group with multifaceted problems in addition to crime, such as substance abuse and various psychological and psychiatric difficulties (1–3). Violent offenders stand out as a particularly important group to reach with risk-reducing interventions due to the destructive consequences of violence on victims and society (4). Persisting violence in high-risk offenders with early onset and complex needs such as personality disorders, substance abuse, and nonviolent criminality (5) is a challenge in forensic settings. Aggressive behaviors often persist in violent offenders with various mental disorders and problems with emotion regulation, impulsivity, and empathy. According to Smeijers et al. (6), research should focus on understanding the reciprocal relations of social information processing, emotions, and emotion regulation in violent offenders. Aggressive behaviors have been related to a lack of social problem-solving skills (7) and emotion dysregulation (8–10), which indicates various skill deficits among this group of offenders. Helping offenders with violent behavior learn anger control and interpersonal problem-solving skills may thus be especially important for reducing the risk for relapse in violent crime (11).

Interventions focused on violent offenders will be affected by target group factors, potentially confounding the treatments’ impact. Such factors may be criminogenic needs, known but not properly addressed in the intervention, such as pro-criminal attitudes (12) and antisocial personality traits (13), but they can also include responsivity factors such as history of trauma (14) and psychiatric problems (3). Impact factors can also be related to how the treatment is facilitated (15), for instance the experience of presence in the virtual environment of Virtual Reality (VR)-assisted treatment (16). To increase the likelihood a treatment is effective and can handle the multifaceted problems among the group, treatment interventions should be based on the principles of risk, need, and responsivity (RNR; (15) for both adults (17, 18) and youth (19, 20). The RNR framework states that offender treatment should target individuals with the highest risk of relapse in crime (risk-principle), focus on dynamic risk factors associated with relapse (needs principle), and be adapted to general evidence of effective treatment and client-specific characteristics [responsivity principle (15)]. Understanding which needs are impacted by treatment and which needs, and responsivity factors impact treatment facilitation is crucial for treatment effectiveness.

Although cognitive behavioral treatment (CBT) programs for offenders has been supported by evidence to be effective in decreasing criminal recidivism (11, 21, 22), offenders’ often complex needs place high demands on individually adapted, yet evidence-based, interventions. Some of the key components of effective treatment for violent offenders are behavioral and skills training (e.g., emotion regulation and social skills) through role-plays based on social problem-solving (22, 23). A recurring challenge in interventions provided in forensic institutions, however, is difficulties with contextual adaptation of such skills training. For both practical and safety reasons, it is difficult to create individually tailored practice situations in the forensic context. Thus, the generalization of skills is currently hampered in such institutions, presumably affecting the offenders’ rehabilitation back to society. There is an urgent need to develop clinical practice in forensic settings, for example, VR technology can provide new opportunities (24).

The use and knowledge of VR as a tool to deliver psychological treatment is developing rapidly in the fields of mental illness and offender rehabilitation. Its effects, which vary in nature and degree, have been demonstrated for several psychiatric disorders such as PTSD, social phobia, schizophrenia, specific phobia, and panic disorder (25). One study focusing on assessment of reactive aggression in students using immersive VR indicated that higher self-reported aggression was correlated to shorter reaction times for aggressive behavior in VR. The VR task was also a better predictor for past violence than self-assessment. This shows promise to VR-assessment in aggression and the authors concluded that future research in the area could be used for clinical samples such as violent offenders (26). VR-assisted treatment for offenders has been described as promising; adding VR as a complementary method to existing treatments creates opportunities for both adapted treatment (27) and controlled research (28). In a recent systematic review, the authors stated that immersive VR-assisted assessment and treatment is feasible and acceptable for offenders, but the evidence for implementing any specific VR intervention remains insufficient (29). In addition to being a tool for interventions, VR is also unique and powerful in creating immersive experiences that can lead to adaptations in responsivity. The two concepts at the heart of understanding the responsive nature of VR are immersion and presence (30). Immersion is best understood as the VR system’s ability to support natural contingencies for perception. Presence is the combination of *place illusion,* the sense of being in the virtual environment, and *plausibility illusion*, the sense that virtual events are actually happening (30, 31). The experiences of spatial presence, involvement, and realness are key factors in measuring presence in an immersive VR experience (16).

Virtual reality-focused research has approached offender treatment from various informative angles. In a study presenting a protocol for aggressive impulse management using the VR-GAIME system (32), Aggression Replacement Therapy (33) was evaluated in a randomized controlled trial with VR added on to impact approach and avoidance behaviors in provoking situations. In VR-GAIME, the participants receive training in decision-making with the task of avoiding disagreeable avatars and approaching agreeable avatars. This study thus aimed at investigating the effect of a motivational intervention on social threat, impacting automatic approach behaviors displayed by individuals high in trait anger (32). Another study investigated criminal expertise (34) using VR, comparing offenders with and without burglary experience with nonoffending community participants, showing that burglars demonstrated a distinct set of burglary skills in relation to the comparison groups. The study could have implications for offender treatment and further reveal the automatic and habitual nature of expertise in decision making (35). Virtual Reality Aggression Prevention Training (VRAPT) is an example of a newly developed VR-assisted treatment (36, 37) aimed to reduce reactive aggression in offenders. The program is CBT based and consists of 16 individual treatment sessions delivered once or twice a week, making the program 8–16 weeks long. VRAPT focuses on skills training in emotion recognition, emotion differentiation, problem-solving, communication, and self-control of impulses and pro-violent cognitions. All sessions in VRAPT but the last include some sort of VR experience. The VR experience in VRAPT is expected to provide; skills training in environments not naturally found in the prison context, more intensive training sessions due to the immersive experience and tailored skills training addressing the needs of the participants to a higher degree than standard CBT-programs for offenders. All in all, the suggestion is that VR has the potential to make offender treatment more precise, intensive and resource efficient. The program starts with an introduction to VRAPT and VR (Session 1), continues with assessment, emotion recognition, and differentiation (Sessions 2–6), skills training in role-play (Sessions 6–15), and ends with an evaluation of the treatment (Session 16). Each session lasts 45–60 min, with 10–40 min per session in VR according to the manual (38, 39). In addition to in-session activities, VRAPT now also includes between-session assignments (40).

Virtual Reality Aggression Prevention Training was evaluated for forensic psychiatric patients in a multicenter RCT (37). The authors showed positive post-treatment effects on self-reported aggression and hostility, anger control skills, anger expression, and impulsiveness, but no effect on staff-reported aggression or long-term effects at the 3-month follow-up. Possible reasons for the non-persistent results, the authors suggest, could be that the model was based on the social information processing model, which does not consider trauma history; that the target group was heterogeneous in psychiatric disorders; that there was a lack of generalization in skills because homework was not assigned, and the scenarios in VR did not match the patients’ everyday life (37). In addition, self-assessment has limitations in a target group with cognitive deficits, behavioral skills were not explicitly measured, and the observational tools may not have been utilized optimally on the wards (37). The authors, however, recommended that future research focus on VR aggression treatment in other forensic populations such as clients in prison with aggressive behavior (37). VRAPT has subsequently been revised to address the initial RCT findings (40). It seems that VR-assisted interventions such as VRAPT can contribute to safer, ecologically valid, and effective interventions for violent offenders in forensic settings. Much work remains, however, to understand how VR-assisted offender treatment should be optimized.

### 1.1. Study design and research questions

The current study is a pilot study of the newly revised VRAPT (40) implemented in a prison setting. The study has a case-series within-group design with pre-treatment, post-treatment, and 12-week post-treatment follow-up measures. The overarching aim is to investigate the impact of VRAPT on key criminogenic needs related to aggressive behaviors, while highlighting important factors that may impact treatment outcomes, with the following specific research questions:

- How do emotion regulation abilities and strategies, aggression, and anger change over time in imprisoned violent offenders participating in VRAPT?
- Which important factors (e.g., experience of presence in the virtual environment, psychosocial background, psychiatric characteristics, pro-criminal attitudes, and prevalence of other externalizing behaviors including substance use) may impact the observed change over time in violent offenders?

## 2. Materials and methods

### 2.1. Sample

Participants were recruited from two medium- and high-security prisons in the Swedish prison and probation service (SPPS). To be included, possible participants had to (1) have a history of violent crime, (2) be sentenced to prison, (3) have been assessed with an increased risk (medium to high) of criminal recidivism, and (4) have an indicated need for treatment of aggression. Aggression was screened using pooled items from the risk and assessment tool Risk, Behov och Mottaglighetsbedömning (RBM_B) (41) with a cutoff value ≥8 indicating the need for treatment. The maximum value for the pooled items was 24, and the within-group range was 8–19 (M = 14, SD = 3.76). Exclusion criteria were (1) inability to understand and provide informed consent, (2) major deficits in understanding the Swedish language preventing active participation, (3) epilepsy, (4) indications of acute psychosis, (5) intellectual disabilities (IQ < 70), (6) acute suicide risk, (7) current and serious security risks preventing safe participation, and (8) less than 10 weeks prison time remaining. The inclusion and exclusion processes were part of regular sentence planning, and investigative staff identified the potential candidates for participation.

A total of 18 male offenders were recruited to the study during the years 2020–2022. Before treatment started, one participant dropped out, and three more dropped out during treatment. Drop-outs were client-initiated (*n* = 3) or administrative (*n* = 1) due to the participant’s sudden transfer to a lower security prison where VRAPT was unavailable. The final sample thus consisted of 14 participants from the high-security (*n* = 6) and medium-security (*n* = 8) prisons. The participants were all violent offenders who had been assessed with a medium (*n* = 2) or high risk (*n* = 12) of relapse to criminality as measured by RBM-B. All participants had violence prevention programs as part of their prison treatment plan and were assessed by the study coordinator (first author DI) as eligible to participate in VRAPT.

Data on (1) current and past aggression, violence, and crime, (2) sociodemographic and psychosocial background, and (3) psychiatric problems, were collected from participants self-reports and structured data collection from file material.

### 2.2. Procedure

#### 2.2.1. Data collection

Data were collected at four time points: (T0) Inclusion screening, (T1) pre-treatment (administered approximately 1 day to 1 week before start of treatment, adjusted after the participants’ possibility to begin), (T2) post-treatment, and (T3) 12-week post-treatment follow-up. See Table 1 for an overview of data collection sources at the different time points. To ensure the reliability of the data, participants were offered support by research staff in answering self-assessment forms. This was done in order to mitigate impact of potential responsivity factors (e.g., impulsivity, reading disabilities, or attention deficits) on answering performance, for example by portioning the text in the questions for better readability.

**Table 1:** Missing data on study measures at data collection points.

Measures	Missing at pre-treatment	Missing at post-treatment, *n*	Missing at follow-up, *n*
*AQ-RSV (Pre-treatment–follow-up)*	N/A	2	3
*DERS (Pre-treatment–follow-up)*	N/A	2	3
*STAXI-2-S (Pre-treatment–follow-up)*	N/A	2	3
*ESI-BF (Pre-treatment)*	N/A	N/A	N/A
*CTQ-SF (Pre-treatment)*	N/A	N/A	N/A
*DSM-XC (Pre-treatment)*	N/A	N/A	N/A
*MCAA—part B (Pre-treatment)*	N/A	N/A	N/A
*IPQ (Post-treatment)*	N/A	2	N/A
*IPQ subscales*	Internal missing, *n*		
*Full scale*	3		
*Spatial presence*	3		
*Involvement*	3		
*Realness*	2		
*Global presence*	3		

#### 2.2.2. Virtual reality aggression prevention training

The mean amount of treatment weeks for the current VRAPT study was 17.5 weeks (SD 11.6, median 13.5), ranging from 7 to 48 weeks. Only half of the VRAPT treatments followed the VRAPT protocol of 8–16 weeks of sessions due to the COVID-19 pandemic, general incidents at the involved prisons, and various logistical reasons. Two of the treatments were shorter than the stipulated protocol (7 weeks) and 5 were longer, ranging 21–48 weeks. All participants received the same number of treatment sessions in accordance with the treatment protocol. Seven program facilitators were trained for the study, each of whom treated 1–5 participants, with a median of two participants per facilitator.

The VR environment was created with the software Social Worlds by CleVR using Oculus rift VR glasses, a high-performance laptop computer, a touch pad for the control of the VR environment, and a microphone and headphones for communicating with the participant in the VR environment. The software included three different functions that were used in VRAPT. The first function was “Walking around,” where the participant could get acquainted with the virtual world. The second function included two parts: “Emotion recognition” and “Emotion differentiation,” where the participant was introduced to avatars displaying different kinds of emotions. The participant was instructed to identify the correct emotions for the avatars. The third and final function that was used was real-time role-play where the program facilitator controlled the avatar’s speech using voice distortion, body language, and emotional responses. Each virtual environment (e.g., park, supermarket, home, office, and prison) had several different situations where the participant could meet between 1 and 3 avatars.

#### 2.2.3. Measures/instruments

Background data (e.g., psychosocial factors, criminal history, risk assessment, description of criminogenic needs, reports of misconduct, and individual plan for ongoing sentence) were collected from SPPS file materials. As a part of file data from RBM-B, we used the Drug Use Disorder Identification Test (42) and the Alcohol Use Disorder Identification Test (43) and the following self-assessment instruments for the different time points from pre-treatment to follow-up (see Table 1).

##### 2.2.3.1. Aggression questionnaire-revised Swedish version

Aggression questionnaire is a self-assessment tool of aggression and hostile behavior containing 29 items spanning over four different factors: Physical aggression (PA), Verbal aggression (VA), Anger (AN), and Hostility (HS) (44). The items in the AQ-RSV version are measured on a five-point Likert scale (1 = least characteristic; 5 = most characteristic) (45). AQ is a highly used self-assessment instrument for aggression and has shown good psychometric properties [global internal consistency: alpha = 0.89 (44); and good generalizability to both the general population (46) and to prison samples (47)]. AQ-RSV has been shown as robust in translation and in a Swedish context (45).

##### 2.2.3.2. Difficulties in emotion regulation scale

Difficulties in emotion regulation scale is a self-assessment tool containing 36 items that measure 6 dimensions of emotion recognition and regulation: Nonacceptance, Goals, Impulse, Awareness, Strategies, and Clarity (48). The items are measured on a five-point Likert scale (1 = almost never, 2 = sometimes, 3 = half the time, 4 = often, and 5 = almost always). The psychometric properties have shown high internal consistency, good test–retest reliability, adequate construct and predictive validity (48), good internal consistency and clinical and predictive utility when used with an adult sample with emotional disorders (49).

##### 2.2.3.3. State–trait anger expression inventory-2-S

State–Trait Anger expression Inventory is a self-assessment of anger, both current and habitual, containing 57 items on the State–Trait anger scale (STAS) and the Anger expression scale (AX) (50). The items are measured on a four-point Likert scale for both State Anger items (1 = not at all, 2 = a little, 3 = rather, and 4 = very) and Trait Anger items (1 = almost never, 2 = sometimes, 3 = often, and 4 = almost always). STAXI-2 is one of the most commonly used instruments for assessing anger (51). The instrument, which is a revision of the STAXI (52), has excellent psychometric qualities in assessing anger (53). The adapted Swedish version, STAXI-2-S, has demonstrated good construct validity and appropriate reliability (54).

##### 2.2.3.4. Externalizing spectrum inventory-brief form

Externalizing spectrum inventory-brief form provides a self-assessment of lifetime externalizing behaviors and contains 160 questions. ESI-BF (55) was developed from the conceptualization of the externalizing spectrum to provide a more fine-grained assessment of impulsiveness/recklessness, substance abuse, and antisocial/aggressive behaviors (56). The items are measured on a four-point Likert scale (1 = true, 2 = partly true, 3 = partly false, and 4 = false) and summarized across the whole scale and on the subscales General Disinhibition (GD), Callous Aggression (CA), and Substance Abuse (SA). The factor structure for the ESI-BF was not confirmed when looking at a sample in a Dutch context, which the authors concluded could be due to cultural differences (56). The criterion validity analysis indicates that the ESI-BF could be more useful as a tool for prediction than as a measurement (56). A study on Swedish forensic psychiatric patients found that ESI-BF showed good to adequate reliability and internal consistency and good criterion validity, but an unclear structural fit (57).

##### 2.2.3.5. Childhood trauma questionnaire-short form

Childhood trauma questionnaire-short form is a self-assessment of traumatic childhood circumstances, containing 28 items covering several types of childhood maltreatment and abuse (58). The items are measured on a five-point Likert scale (1 = never true, 2 = seldom true, 3 = sometimes true, 4 = often true, and 5 = very often true) and organized into five trauma types: emotional abuse, physical abuse, sexual abuse, emotional neglect, and physical neglect, which are assessed as either none (minimal); low to moderate; moderate to severe; or severe to extreme (58). CTQ is a well-established test of childhood trauma showing good psychometric properties (59), with research needed on test–retest reliability, measurement error, and criterion validity (60). CTQ demonstrates a strong level of evidence regarding adequate internal consistency, reliability, content validity, structural validity, and convergent validity, with CTQ-SF as a good alternative (61).

##### 2.2.3.6. DSM-5 self-rated level 1 cross-cutting symptom measure

DSM-5 self-rated level 1 cross-cutting symptom measure provides a 23-item self-assessment of 13 domains of mental illness important to psychiatric diagnostics (62). Each item focuses on how often during the last 2 weeks; the participant has been bothered by the symptoms. The items are measured on a five-point Likert scale (0 = none or not at all; 1 = slight or rare, less than a day or two; 2 = mild or several days; 3 = moderate or more than half the days; and 4 = severe or nearly every day). When using DSM-XC, a rating of mild (i.e., 2) or greater on any item in 10 of the domains (depression, anger, mania, anxiety, somatic symptoms, sleep problems, memory, repetitive thoughts and behaviors, dissociation, and personality functioning) may guide decisions about additional assessments (62). For substance use, suicidal ideation, and psychosis, a rating of slight (i.e., 1) or greater on any item within the domain may serve the same purpose (62). The psychometric properties of DSM-XC were evaluated through the field trials of DSM-5 (63–65) and found to have adequate test–retest reliability for all items except the two on mania (64). When evaluated as a screening tool on a sample of healthy adults, the conclusion was that the DSM-XC proved to have a good specificity (66). However, DSM-XC was not developed as a screening tool; it can, however, be a good instrument for transdiagnostic assessment in research and clinical use (66).

##### 2.2.3.7. Measures of criminal attitudes and associates part B

Measures of criminal attitudes and associates gives a self-assessment of pro-criminal attitudes, containing 46 items measured across the areas of Violence, Entitlement, Antisocial Intent, and Attitudes Toward Associates (12). The items are measured on a four-point Likert scale (1 = disagree, 2 = undecided, 3 = agree, and 4 = agree completely) in the Swedish version of the questionnaire (67). The scale has shown acceptable levels of reliability and validity in a sample of incarcerated males (12), and when translated to Swedish and evaluated with an offender sample and a public sample, it showed satisfactory psychometric properties (67). MCAA has shown good predictive validity for relapse to both general and violent crime (68), and it is useful in understanding the dynamic risk factor of criminal attitudes (12, 68).

##### 2.2.3.8. Igroup presence questionnaire

Igroup presence questionnaire, used as a self-assessment of presence in the virtual environment, contains 14 items across three subscales: Spatial presence, Involvement, and Realness (16). The items are measured on a seven-point Likert scale, with higher ratings indicating a higher degree of experienced presence in VR. IPQ has shown good internal consistency across several translations (16, 69, 70).

### 2.3. Statistical analysis

R (version 4.2.1) was used for all statistical analysis. We analyzed change over time using robust Bayesian linear mixed effects models, with participant ID as the random effect. All Bayesian statistical models were specified using the R package brms (71), interfacing R with the Stan probabilistic programming language (72). Robustness was achieved using Student *t* likelihood (73), which alleviates the impact of potential outliers. Furthermore, all priors were chosen to be weakly informative and to thus having negligible impact on obtained estimates while still providing moderate regularization (74). Model sampling using Markov Chain Monte Carlo (MCMC) was conducted using four chains with 4,000 iterations each. All models converged well, with Gelman-Rubin diagnostics (R-hat) of 1.00 (75).

Results from Bayesian analyses are presented as the median posterior estimate of change between the time points, with the associated 90% highest density interval (HDI) presented within square brackets. The 90% HDI may be interpreted such that it has a 90% probability of containing the actual value. Since there is no notion of statistical significance in Bayesian statistics, we followed guidelines suggesting that a probability of 90% or higher can be considered very likely (76). We therefore considered an estimated change as robust if the 90% HDI did not contain zero and was very likely different from zero. In addition, we also calculated the probability of direction (*PD*), which is the probability, ranging from 50 to 100%, that the estimated change is either positive or negative (77). Since we were interested in lowered scores for all outcomes, we present the probability of the estimated change being negative. The *PD* has a 1:1 numerical correspondence with frequentist *p* values such that *P*two − sided = 2 × (1 − *PD*).

We investigated the impact of potential confounding factors separately by including each potential confounder as a covariate for each outcome. The leave-one-out cross-validated expected log predictive density (ELPD_LOO_) (78); was then used to quantify and compare model fit. The relative model fit measure ELPD_LOO_ provides an estimate of predictive accuracy for the model’s out-of-sample fit compared to another model fit on the same data, but with a different set of variables. We multiplied obtained values by 1, so that lower values of ELPD_LOO_ indicated better model fit. Differences in ELPD_LOO_ of <4 points are generally considered unreliable and of no clear predictive advantage, thus favoring the least complex model (79).

Finally, we conducted supplementary analysis using the reliable change index (RCI) developed by Jacobson and Truax (80) to further explore the direction of individual change. RCI is the individual post-treatment or follow-up measurement subtracted by the individual pre-treatment measurement divided by the standard error of the change between measures and is calculated by the following formulas:


$$RCI=\frac{X2-X1}{S_{\mathrm{diff}}}$$

$$S_{\mathrm{diff}}=\sqrt{2{\left(S_{E}\right)}^{2}}$$

Standard error (SE) was calculated by multiplying the standard deviation for a normal population times the square of 1 – the instrument’s internal consistency:


$$SE=S_{1}\sqrt{1-r_{xx}}$$

According to the authors, a reliable change beyond ±1.96 is unlikely with an alpha level of *p* < 0.05 without a real change, therefore this indicates reliable change. The best cutoff for change indicating a move from a pathological level to a normal level uses the C criteria (80). Cutoff C is calculated using the SD for the clinical group multiplied by the mean for the normal population and adding this with the SD for the normal population multiplied by the mean for the clinical group divided by the added SDs from the normal population and clinical group. The calculation uses the following formula:


$$\mathrm{CutoffC}=\frac{\left(SD_{\mathrm{clinical}}xM_{non-\mathrm{clinical}}\right)+\left(SD_{non-\mathrm{clinical}}xM_{\mathrm{clinical}}\right)}{\left(SD_{\mathrm{clinical}}+SD_{non-\mathrm{clinical}}\right)}$$

Based on RCI and cutoff, participants were divided into the categories *Recovered* (those who passed the cutoff and made a reliable change), *Improved* (those who did not pass the cutoff but made reliable change), *Unchanged* (neither passed the cutoff nor made a reliable change), and *Deteriorated* (made a reliable change, but in the wrong direction) (80). Participants below cutoff at pre-treatment, but who showed a reliable change were assigned to the *Improved* category. Missing values at post-treatment and follow-up assigned the participant to the *Unchanged* category. Due to missing data, reliable change was true for different participants for different time points.

## 3. Results

### 3.1. Sample characteristics

The mean age of the participants was 29 years (SD = 8.1, median = 28.5, range 20–49). The majority (71%, *n* = 10) were 20–30 years old. Most (64%, *n* = 9) had a high school degree, while 21% (*n* = 3) lacked that qualification. Two participants had a degree from senior high school. Fewer than half (43%, *n* = 6) owned their own homes at the time of imprisonment, and an equal number (43%, *n* = 6) were homeless. Two participants had temporary living conditions outside of prison. Almost all participants (93%, *n* = 13) had no work outside prison; only one worked or studied part-time. Foster home placement prior to age 18 was uncommon (valid for only 21%, *n* = 3). Repeated misbehavior before age 15 was, however, common (57%, *n* = 8), with occasional misbehavior being the second most common category (21%, *n* = 3), followed by mainly well-behaved (14%, *n* = 2) and unknown (7%, *n* = 1).

For self-reported mental health, overall and across all DSM-XC domains, 71% (*n* = 10) of participants were eligible for some sort of additional assessment. The number of domains above the cutoff for additional assessment ranged 1–11 for the whole sample, with a median of four domains above cutoff per participant. The most common domains above cutoff were depression and mania (50%, *n* = 7). Four participants had at least one domain reported as severe, with a range of 1–8 domains. See Table 2 for individual variety across domains.

**Table 2:** DSM-XC variety in domains.

DSM-XC domains	None, *n*	Slight, *n*	Mild^*^, *n*	Moderate, *n*	Severe, *n*	Total, *N*	Above cutoff, %
*Depression*	5	2	N/A	4	3	14	50
*Anger*	2	6	2	1	3	14	43
*Mania*	4	3	2	5	N/A	14	50
*Anxiety*	6	3	4	N/A	1	14	36
*Somatic symptoms*	7	3	1	2	1	14	29
*Sleep problems*	5	3	3	2	1	14	43
*Memory*	9	2	3	N/A	N/A	14	21
*Repetitive thoughts and behaviors*	8	3	1	1	1	14	21
*Dissociation*	7	5	N/A	N/A	2	14	14
*Personality functioning*	10	2	N/A	1	1	14	14
DSM-XC domains	None	Slight^**^	Mild	Moderate	Severe	Total	Above cutoff
*Substance use*	N/A	N/A	N/A	N/A	N/A	14	N/A
*Suicidal ideation*	N/A	N/A	N/A	N/A	N/A	14	N/A
*Psychosis*	N/A	N/A	N/A	1	N/A	14	7%

^*^Cutoffs indicate depression, anger, mania, anxiety, somatic symptoms, sleep problems, memory, repetitive thoughts and behaviors, dissociation, personality functioning. ^**^Cutoffs indicate substance use, suicidal ideation, and psychosis.

File data on mental health showed that 50% (*n* = 7) had a diagnosis of mental disorder, with the most common being ADHD (43%, *n* = 6). Other indexed diagnoses were personality disorder (7%, *n* = 1), personality disorder due to organic brain damage (7%, *n* = 1), post-traumatic stress syndrome (7%, *n* = 1), and depression (7%, *n* = 1). In addition, various undiagnosed aspects of mental illness registered were anxiety and panic attacks, depressive mood, recurring nightmares, and a history of suicidal ideation. A total of 36% (*n* = 5) had no registered mental illness, and 14% (*n* = 2) had unspecified (depressive, anxiety) mental illness. Most of the group (79%, *n* = 11) had no history of suicide attempts and none of the participants had attempted suicide within the last year. Diagnoses of substance abuse disorders (29%, *n* = 4) and alcohol abuse disorder (7%, *n* = 1) were uncommon in the sample. However, 64% (*n* = 9) of the participants scored >0 on the DUDIT measure (81), with a mean of 20 (SD = 13.03 and median = 17) and a range of 3–40. A score of <6 on the DUDIT is an indication of substance use problems, and a score of ≥25 indicates that substance abuse syndrome is probable (82). The most common drug used in the entire sample was cannabis (64%, *n* = 9), followed by cocaine (43%, *n* = 6). Many participants had a history of using various drug (57%, *n* = 8). A total of 64% (*n* = 9) scored >0 on the AUDIT measure (83) with a mean of 8.1 (SD = 5.37; median = 10) and a range of 1–18. The mean for the subsample is in the zone indicating risky alcohol consumption (a score of 8–15) and the range carries over to the zone of problematic alcohol consumption (a score of 16–19) (82).

The participants’ index crimes comprised 1–8 different offenses, covering a total of 31 different categories of offenses (e.g., attempted murder, murder, robbery, aggravated robbery, drunk driving, theft, and minor and major drug offenses). The most common index crime was robbery. The length of prison sentence ranged from 7 months to life imprisonment, and the range of prior prosecutions was 1–25 (M = 10.78, median = 10). For a summary of the participants’ additional antisocial history and behaviors, see Table 3.

**Table 3:** Antisocial history and behaviors.

Antisocial history and behaviors (*N* = 14)			
*Family members convicted of a violent crime: n*	≥2: 3	1: 2	0: 9
*Prior SPPS sentence: n*	Yes: 9	No: 5	
*Prior prosecutions: n*	≥2: 9	1: 3	0: 2
*violent crimes: n*	≥2: 8	1: 2	0: 4
*Age of 1st prosecution, years: n*	<18: 8	18–20: 3	>21: 3
*-age of first prosecution for violent offense, years: n*	<18: 3	18–20: 4	>21: 7
*Institutionalizations for misconduct: n*	>2: 2	1: 6	0: 6

Misconduct during imprisonment was measured both before and during the VRAPT trial. A large proportion of the sample (79%, *n* = 11) had reported or suspected acts of misconduct prior to the pre-treatment measure, with ranges of 0–10 for reported misconduct (M = 3, SD = 3.11) and 0–8 for suspected misconducts (M = 2.85, SD = 2.68). Together, the range of reported and suspected misconducts was 0–18 (M = 5.85, SD = 5.05). No criminal acts were committed during imprisonment, and the number of participants with reports of violence (14%, *n* = 2) or suspected violence (14%, *n* = 2) was small. One participant, however, was responsible for nine acts of reported violence prior to the pre-treatment measure and another had three acts of suspected violence.

Externalizing behaviors, childhood adverse events, and pro-criminal attitudes as measured by the ESI-BF, CTQ-SF, and MCAA are presented in Table 4.

**Table 4:** Outcome measures and impacting factors at pre-treatment for the whole sample (*N* = 14).

Measures	Mean	SD	Range
AQ-RSV	94.4	23.4	39–124
DERS	92.9	23.5	44–120
STAXI-State	24	11.9	15–51
STAXI-Trait	24.7	8.1	11–39
STAXI-AX	53.9	17.9	13–76
ESI-BF	254.4	71	110–343
General disinhibition	36	12.7	13–51
Callous aggression	31.4	9.2	15–44
Substance abuse	25.9	8.9	12–38
CTQ-SF	60.5	6.9	50–72
Emotional abuse	8.1 (none–low)	3.7	5–15 (none–moderate)
Physical abuse	9.5 (low–moderate)	3.9	5–16 (none–severe)
Sexual abuse	-	-	-
Emotional neglect	17.1 (moderate–severe)	4.9	8–25 (none–severe)
Physical neglect	13 (severe)	2	10–17 (low–severe)
MCAA	128.1	29.4	70–160

#### 3.1.1. Time spent and presence in the virtual environment

The mean time spent in the virtual environment in a session was estimated by the participants as 24.5 min (range 10–45, SD = 10.12). Because the experience of presence as measured by the IPQ was severely skewed, we used the median and interquartile range (IQR) for this measure. The results were highly variable and ranged from −1.5 to 6. Average score for spatial presence was 6 (IQR = 0.5–8), general presence 2 (IQR = 1–3), involvement 1 (IQR = −4.5–4.5), and experienced realism −1.5 (IQR = −3–2).

### 3.2. Change in aggression, emotion regulation, and anger following VRAPT

#### 3.2.1. Aggression

**Figure 1:**
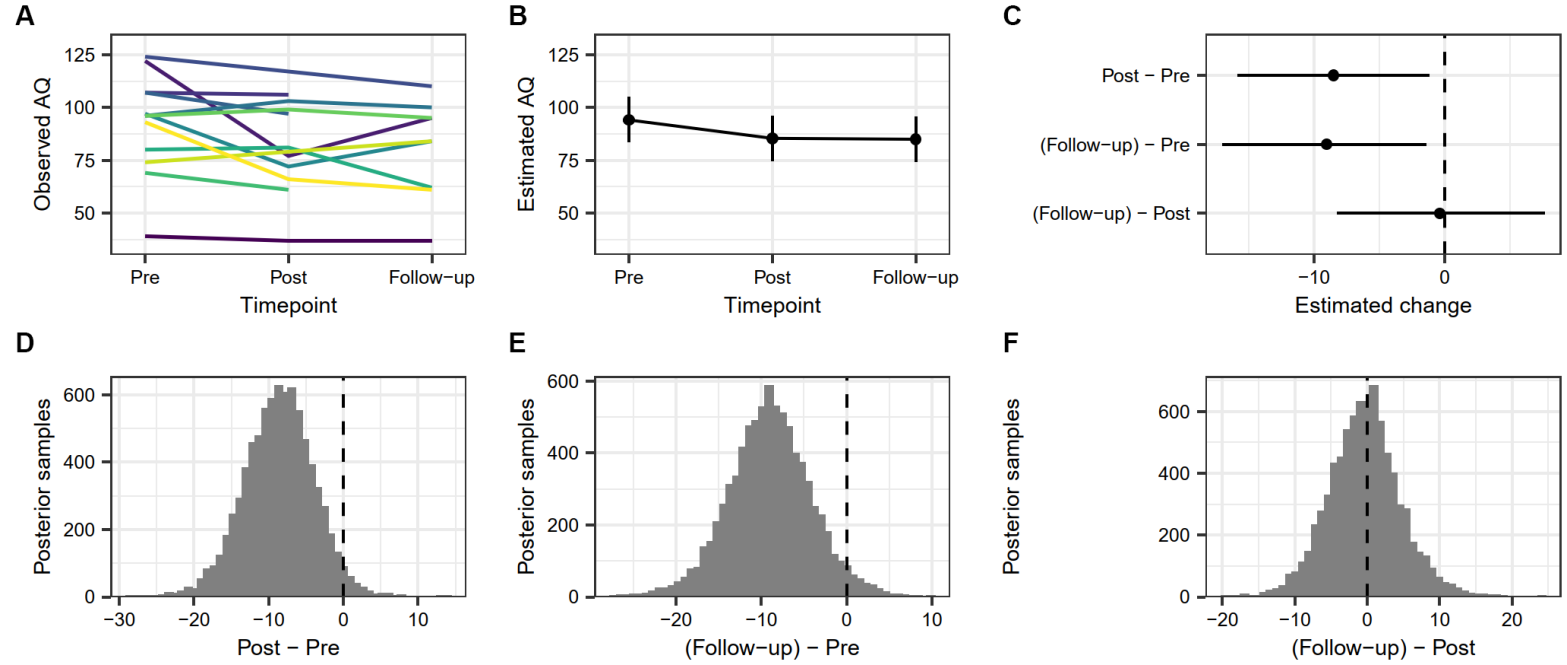
**(A)** Individual trajectories showing observed values of AQ-RSV score across timepoints. **(B)** Model-based estimate of AQ-RSV score across timepoints, with associated 90% highest density interval. **(C)** Model-based change in AQ-RSV score between timepoints, with associated 90% highest density interval. **(D)** Posterior distribution of estimated difference in AQ-RSV score from pre-treatment to post-treatment. **(E)** Posterior distribution of estimated difference in AQ-RSV score from pre-treatment to follow-up. **(F)** Posterior distribution of estimated difference in AQ-RSV score from post-treatment to follow-up.

The estimated change in AQ-RSV scores from pre-treatment to post-treatment was 8.5 [1.16, 15.88], and the estimated change from pre-treatment to follow-up was 9.03 [1.4, 17.05]. There was no robust difference between post-treatment and follow-up (0.38 [−7.67, 8.25]). The probability that AQ-RSV scores were lower at post-treatment than at pre-treatment was 97.45%, and the probability that AQ-RSV scores were lower at follow-up than at pre-treatment was 96.74%. Figure 1 demonstrates changes in aggression as measured by AQ-RSV for the whole sample over all measure points.

The AQ-RSV trajectories of individual participants from pre-treatment to follow-up can be described as either decreasing at each measure point (*n* = 2), increasing at each measure point (*n* = 1), or showing variability in increase and decrease (*n* = 6). Two participants lacked post-treatment data but showed a general, decreasing curve, while three others also lacked follow-up data, but showed a decreasing trend between pre-treatment and post-treatment.

Three participants showed higher aggression levels at follow-up than at pre-treatment. The rest of the sample (*n* = 11) had either lower aggression at follow-up than at pre-treatment (*n* = 8) or incomplete data for follow-up (*n* = 3). Stated differently, 73% of the participants with valid data showed decreased aggression between pre-treatment and follow-up.

Reliable change (RC) between pre-treatment and post-treatment as indicated by a value greater than ±1.96 was true for seven participants with complete pre-treatment and post-treatment measures. Three of these participants had a post-treatment value under the calculated cutoff value (84.7), indicating a status of recovered, and three showed an RC but did not cross the cutoff, indicating an improved status. One participant was in the Deteriorated category. The rest of the sample (*n* = 7) was unchanged. See Table 5 for complete RC data between pre-treatment to post-treatment.

**Table 5:** Reliable change and cutoffs on outcome measures between pre- and post-treatment.

Measure	RC >±1.96^*^	Cutoff	Recovered, *n* (%)	Improved, *n* (%)	Unchanged^***^, *n* (%)	Deteriorated *n* (%)	Below cutoff at pre-treatment *n* (%)
AQ-RSV	−17.3–2.9	84.7	3 (21)	3 (21)	7 (50)	1 (7)	4 (29)
DERS	−30.7–3.0	85	4 (29)	5 (36)	4 (29)	1 (7)	4 (29)
STAXI-Trait	−15.2–(−2.2)	20.9	3 (21)	7 (50)	4 (29)	N/A	4 (29)
STAXI-AX	−24.5–3.2	42.2	6 (42)	4 (29)	3 (21)	1 (7)	3 (21)

^*^SE and S_diff:_ AQ-RSV = 1.85 and 2.62 (IC = 0.89^**^), DERS = 1.45 and 2.05 (IC = 0.93^**^), STAXI-Trait = 0.65 and 0.92 (IC = 0.88^**^), and STAXI-Anger Expression Index = 1.57 and 2.2 (IC = 0.88^**^). ^**^AQ-RSV (44), DERS (48), and STAXI-2 (50). ^***^Missing Post-treatment data: *n* = 2.

Reliable change from pre-treatment to follow-up was true for eight participants with complete pre-treatment and follow-up data. Of these, 2 were classified as Recovered, 4 as Improved, and 1 as Deteriorated. The rest of the sample was Unchanged (*n* = 7). See Table 6 for complete AQ-RSV RC data from pre-treatment to follow-up.

**Table 6:** Reliable change and cutoffs on outcome measures between pre-treatment and follow-up.

Measure	RC >±1.96^*^	Cutoff	Recovered *n* (%)	Improved *n* (%)	Unchanged^***^ *n* (%)	Deteri-orated *n* (%)	Below cutoff at pre-treatment *n* (%)
AQ-RSV	−10.3–8.4	84.7	2 (14)	4 (29)	7 (50)	1 (7)	4 (29)
DERS	−18.5–2.9	85	4 (29)	4 (29)	5 (36)	1 (7)	4 (29)
STAXI-trait	−11.9–5.4	20.9	3 (21)	3 (21)	7 (50)	1 (7)	4 (29)
STAXI-AX	−16.4–5	42.2	4 (29)	4 (29)	5 (36)	1 (7)	3 (21)

^*^SE and S_diff:_ AQ-RSV = 1.85 and 2.62 (IC = 0.89^**^), DERS = 1.45 and 2.05 (IC = 0.93^**^), STAXI-Trait = 0.65 and 0.92 (IC = 0.88^**^), STAXI-Anger Expression Index = 1.57 and 2.2 (IC = 0.88^**^). ^**^AQ-RSV (44), DERS (48), and STAXI-2 (50). ^***^Missing Follow-up data: *n* = 3.

#### 3.2.2. Difficulties in emotion regulation

**Figure 2:**
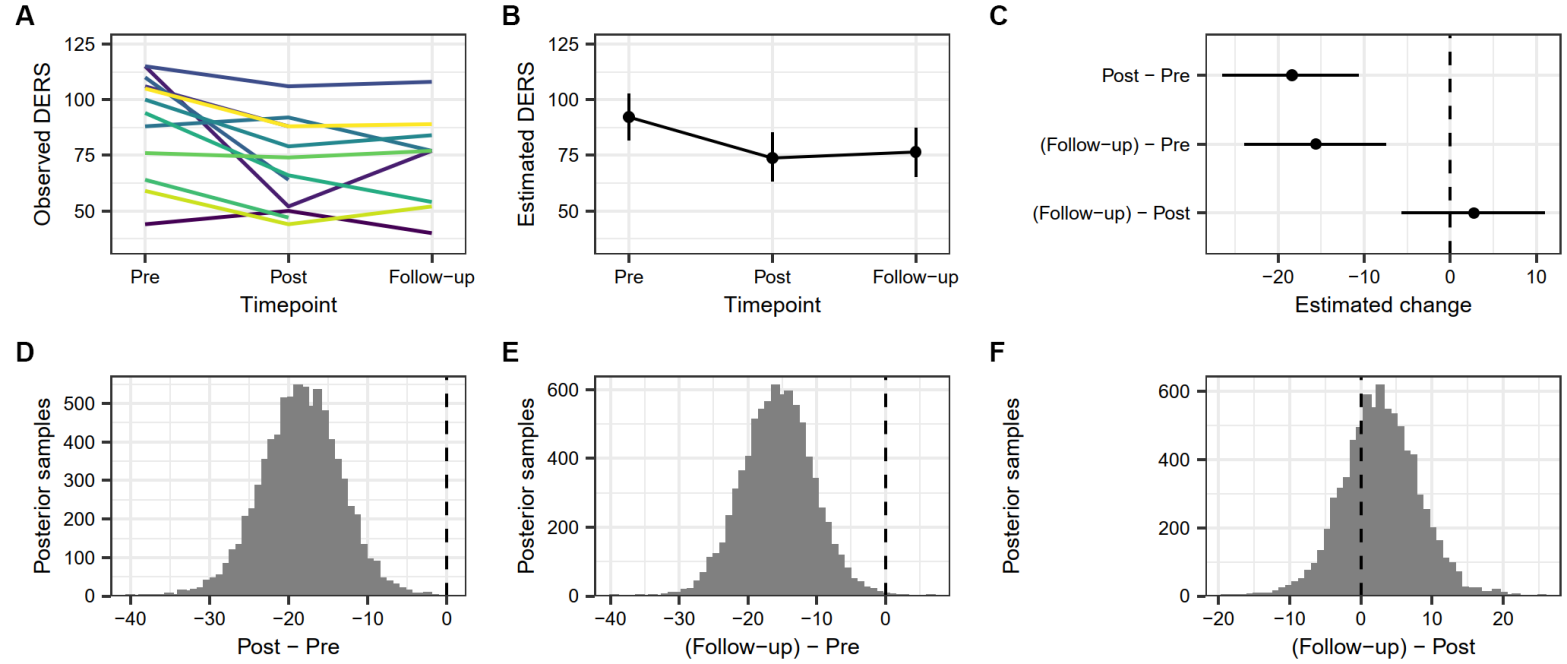
**(A)** Individual trajectories showing observed values of DERS score across timepoints. **(B)** Model-based estimate of DERS score across timepoints, with associated 90% highest density interval. **(C)** Model-based change in DERS score between timepoints, with associated 90% highest density interval. **(D)** Posterior distribution of estimated difference in DERS score from pre-treatment to post-treatment. **(E)** Posterior distribution of estimated difference in DERS score from pre-treatment to follow-up. **(F)** Posterior distribution of estimated difference in DERS score from post-treatment to follow-up.

The estimated change in DERS score from pre-treatment to post-treatment was 18.37 [10.65, 26.53], and the estimated change from pre-treatment to follow-up was 15.6 [7.39, 23.94]. There was no robust difference between post-treatment and follow-up (−2.76 [−11.02, 5.67]). The probability that DERS scores were lower at post-treatment than at pre-treatment was 99.99%, and the probability that DERS scores were lower at follow-up than at pre-treatment was 99.89%. Change in emotion regulation difficulties for the sample as measured by DERS is demonstrated in Figure 2.

Different individual trends between pre-treatment to follow-up were evident, with emotion dysregulation either decreasing at every measure point (*n* = 1) or showing variability in increase and decrease (*n* = 8). Of two participants lacking post-treatment data, one showed a decrease and the other an increase in emotion dysregulation from pre-treatment to follow-up, and three others lacked follow-up data but showed a decreasing trend from pre-treatment to post-treatment. Overall, two participants showed an increased level of emotion dysregulation from pre-treatment to follow-up. The rest of the sample (*n* = 12) had either incomplete data at follow-up (*n* = 3) or lower (*n* = 9) emotion dysregulation at follow-up than at pre-treatment. Thus, approximately 82% of the participants with valid follow-up data showed a decrease in emotion dysregulation from pre-treatment to follow-up.

Reliable change between pre-treatment and post-treatment as indicated by a score greater than ±1.96 was true for 10 participants with complete pre-treatment and post-treatment measures. Four of these participants had a post-treatment value under the calculated cutoff (84), indicating a Recovered status and five had a RC value above cutoff, indicating an Improved status. One participant had a Deteriorated status from pre-treatment to post-treatment. The rest of the sample (*n* = 4) was Unchanged. See Table 5 for complete data on DERS RC between pre-treatment and post-treatment.

Reliable change from pre-treatment to follow-up was true for nine participants with complete pre-treatment and follow-up measures. Of these, four were classified as Recovered, four as Improved, and one as Deteriorated. The rest of the sample was Unchanged (*n* = 5). See Table 6 for complete data on DERS RC between pre-treatment and follow-up.

#### 3.2.3. Anger

##### 3.2.3.1. State anger

Due to the low variance in the STAXI-State score, this subscale could not be used in the mixed effects models and was therefore left out of the analysis on probability of change between time points. For consistency, the STAXI-State score was also kept out of the RCI analyses.

Between pre-treatment and post-treatment, anger scores decreased at every time point (*n* = 1), varied between increasing and decreasing (*n* = 6), or showed no variability at all (*n* = 2). Of the three participants who lacked follow-up data, two showed a decrease in anger from pre-treatment to post-treatment, and one showed no trend. Of the two participants who lacked post-treatment data, both showed no trend between pre-treatment and follow-up. At follow-up, the sample had either incomplete data (*n* = 3), increased state anger (*n* = 1), no change (*n* = 3), or decreased state anger (*n* = 5) since pre-treatment. In other words, 45% of the sample with valid follow-up data showed decreased levels of state anger between pre-treatment and follow-up.

##### 3.2.3.2. Trait anger

**Figure 3:**
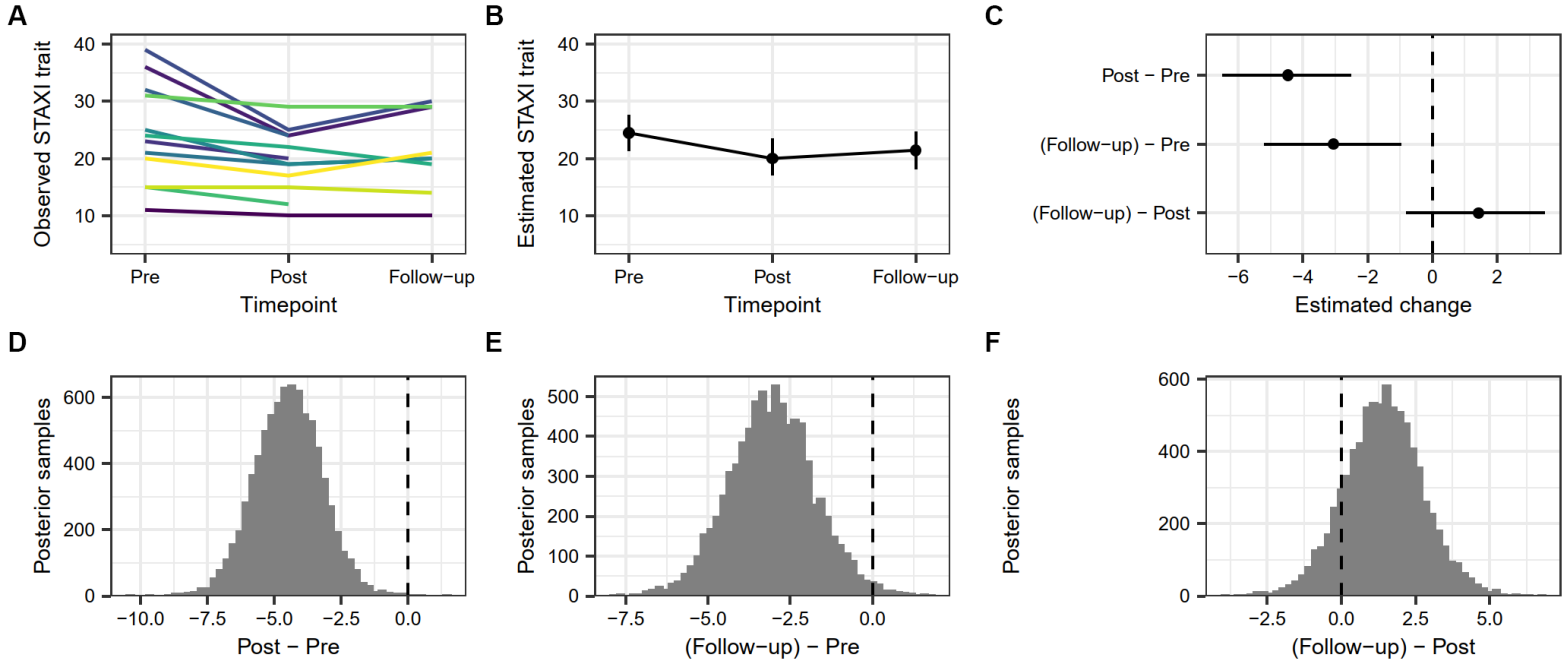
**(A)** Individual trajectories showing observed values of STAXI-Trait score across timepoints. **(B)** Model-based estimate of STAXI-Trait score across timepoints, with associated 90% highest density interval. **(C)** Model-based change in STAXI-Trait score between timepoints, with associated 90% highest density interval. **(D)** Posterior distribution of estimated difference in STAXI-Trait score from pre-treatment to post-treatment. **(E)** Posterior distribution of estimated difference in STAXI-Trait score from pre-treatment to follow-up. **(F)** Posterior distribution of estimated difference in STAXI-Trait score from post-treatment to follow-up.

The estimated change in STAXI-Trait score from pre-treatment to post-treatment was 4.46 [2.51, 6.51], and the estimated change from pre-treatment to follow-up was 3.05 [0.97, 5.21]. There was no robust difference between post-treatment and follow-up (−1.43 [−3.48, 0.81]). The probability that STAXI-Trait scores were lower at post-treatment than at pre-treatment was 99.95%, and the probability that STAXI-Trait scores were lower at follow-up than at pre-treatment was 98.92%. Figure 3 demonstrates changes in trait anger as measured by STAXI-trait for the whole sample over all measure points.

Between pre-treatment and follow-up, members in the sample were either decreasing in trait anger at every measure point (*n* = 1) or showed variability in increase and decrease (*n* = 8). Three participants lacked follow-up data, and all of them showed a decrease between pre-treatment and post-treatment. Two participants lacked post-treatment data, with one showing an increasing trend and the other a decreasing trend between pre-treatment and follow-up. The sample had either incomplete data for follow-up (*n* = 3) or increased (*n* = 2) or decreased (*n* = 9) levels of state anger at follow-up than at pre-treatment. In other words, 64% of the sample with valid follow-up data showed decreased levels of state anger between pre-treatment and follow-up.

Reliable change between pre-treatment and post-treatment as indicated by a score greater than ±1.96 was true for 10 participants with complete pre-treatment and post-treatment measures. Three of these showed a change below the cutoff (20.9), indicating a Recovered status after the treatment, and seven showed a change that did not drop below the cutoff, indicating an Improved status. The rest of the sample (*n* = 4) was Unchanged. All the unchanged participants were under the calculated cutoff at pre-treatment. No participant deteriorated in trait anger between pre-treatment and follow-up. See Table 5 for complete RC data between pre-treatment and post-treatment.

Reliable change between pre-treatment and follow-up was true for seven participants with complete pre-treatment and follow-up measures. Of these, three participants were classified as Recovered, three as Improved, and one as Deteriorated. The rest of the sample was Unchanged (*n* = 7). See Table 6 for complete RC data between pre-treatment and follow-up.

##### 3.2.3.3. Anger expression

**Figure 4:**
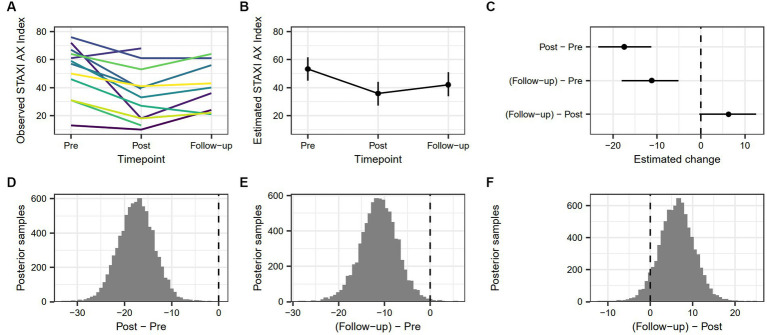
**(A)** Individual trajectories showing observed values of STAXI AX Index score across timepoints. **(B)** Model-based estimate of STAXI AX Index score across timepoints, with associated 90% highest density interval. **(C)** Model-based change in STAXI AX Index score between timepoints, with associated 90% highest density interval. **(D)** Posterior distribution of estimated difference in STAXI AX Index score from pre-treatment to post-treatment. **(E)** Posterior distribution of estimated difference in STAXI AX Index score from pre-treatment to follow-up. **(F)** Posterior distribution of estimated difference in STAXI AX Index score from post-treatment to follow-up.

The estimated change in STAXI-AX Index score from pre-treatment to post-treatment was 17.43 [11.28, 23.39], and the estimated change from pre-treatment to follow-up was 11.18 [5.08, 18.02]. There was no robust difference between post-treatment and follow-up (−6.3 [−12.58, 0.37]). The probability that STAXI-AX Index scores were lower at post-treatment compared to pre-treatment was >99.99%, and the probability that STAXI-AX Index scores were lower at follow-up compared to pre-treatment was 99.75%. Figure 4 demonstrates changes in anger expression as measured by STAXI-AX Index for the whole sample over all measure points.

Different trends between pre-treatment and follow-up in the sample showed a decrease every measure point (*n* = 1) or variability between increase and decrease (*n* = 8). Of the three participants who lacked follow-up data, two showed a decrease between pre-treatment and post-treatment, and a single participant showed an increasing trend. Two participants lacked post-treatment data but showed a decreasing trend from pre-treatment to follow-up. The sample had incomplete data for follow-up (*n* = 3), increased state anger (*n* = 1), no change (*n* = 2), or decreased state anger (*n* = 8) between pre-treatment and follow-up. In other words, 57% of the sample with valid follow-up data showed decreased levels of anger expression between pre-treatment and follow-up.

Reliable change between pre-treatment and post-treatment as indicated by a score greater than ±1.96 was true for 11 participants with complete pre-treatment and post-treatment measures. Six of these showed a change below the cutoff (42.2), indicating a Recovered status post-treatment, four showed no change below cutoff and were Improved, and one participant was Deteriorated. The rest of the sample (*n* = 3) was Unchanged. Three participants were under the calculated cutoff at pre-treatment. See Table 5 for complete RC data.

Reliable change between pre-treatment and follow-up was true for nine participants with complete pre-treatment and follow-up measures. Of these, four participants were Recovered, four were Improved, and one was Deteriorated. The rest of the sample was Unchanged (*n* = 5). See Table 6 for complete RC data between pre-treatment and follow-up.

### 3.3. Possible factors impacting outcome from VRAPT

**Figure 5:**
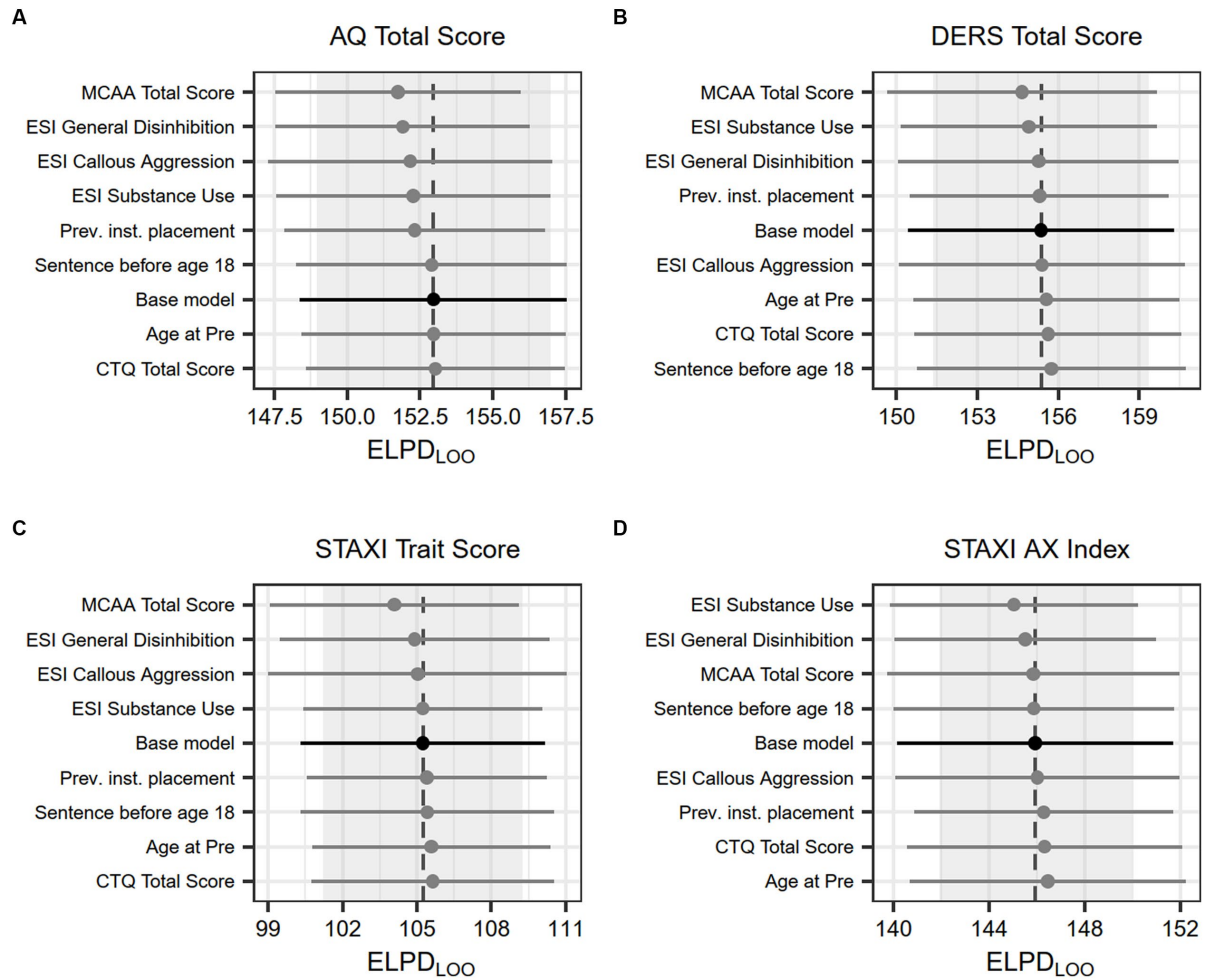
Effect of confounding factors on model fit. Dots show the average leave-one-out cross-validated expected log predictive density, lines show the associated standard error. A difference of four or less is considered unreliable and of no clear predictive advantage, thus favoring the base model.

Analysis of potential confounding factors revealed no impact on the model’s predictive performance for any confounding factor, regardless of outcome, with none of the ELPD_LOO_ values showing a change of at least **four** points. The base model, without any impacting factors, thus exhibited the best predictive performance. Results are visualized in Figure 5.

Reliable change analyses identified participants as either Recovered, Improved, Unchanged, or Deteriorated. In terms of impacting factors, the group that Deteriorated on at least one outcome measure (*n* = 4, 29% of N) was assessed as interesting for further exploratory analysis. The Deterioration group showed lower values across all measures except STAXI-State anger (equal) and ESI-BF SA and CTQ-SF (both higher). The CTQ-SF subscales showed that the Deterioration group had less experience of physical abuse (none–low) and more experience of emotional and physical neglect (moderate–severe) than the rest of the sample. See Table 7 for outcome measures and factors impacting the deterioration group.

**Table 7:** Outcome measure and impacting factors for the deterioration group (*n* = 4).

Measures	Mean	SD	Range
AQ-RSV	84.2	30.6	39–107
DERS	89.5	33.0	44–120
STAXI—state	24	18.0	15–51
STAXI—trait	20.5	6.8	11–27
STAXI—AX	48	23.4	13–61
ESI-BF	250.2	94.4	110–311
General disinhibition	33.8	14.4	13–46
Callous aggression	27.3	10.0	15–39
Substance abuse	29.3	11.7	12–38
CTQ	65.3	4.6	60–70
Emotional abuse	7.5 (none)	5.0	5–15 (none–moderate)
Physical abuse	8.3 (none–low)	2.5	5–11 (none–low)
Sexual abuse	5 (none)	N/A	5–5 (none)
Emotional neglect	20.3 (severe)	5.0	15–25 (moderate–severe)
Physical neglect	15 (severe)	1.8	13–17 (severe)
MCAA	123.2	37.4	70–153

## 4. Discussion

In this pilot study on violent offenders undergoing VRAPT treatment, we could estimate that a decrease in emotion dysregulation, aggression, and trait anger as well as anger expression was very likely (> 95% probability across all outcome measures) to occur between pre-treatment to post-treatment and follow-up. We could also show RC at a Recovered or Improved level for just under half or slightly more than two-thirds of the sample, respectively. However, no robust difference was demonstrated between post-treatment and follow-up and no robust impact of potential impacting factors on the model were demonstrated for any of the outcome measures.

Considering the first research question, “How do emotion regulation abilities and strategies, aggression, and anger change over time in imprisoned, violent offenders participating in VRAPT?” RC analyses revealed that 42% (6/14) of the sample recovered or improved in terms of aggression and as many as 65% (9/14) either recovered or improved in terms of emotion regulation between pre-treatment and post-treatment. For trait anger 71% (10/14) either recovered or improved, which was also true for anger expression. RC analyses of the period between pre-treatment and follow-up produced similar results, but with fewer participants showing RC, recovery, or improvement, and more participants staying unchanged, in line with the upward slope trend between post-treatment to follow-up shown in Figures 1–4. Consistently across outcome measures, a majority of the unchanged group was below the calculated cutoff C for RC at pre-treatment, possibly indicating a floor effect. RNR-based research states that individuals with higher risk for relapse tend to benefit more from treatment (15, 85) and that offender treatment should focus on the appropriate criminogenic needs of the individual (15). The sample consisted of offenders with a medium to high risk of relapse into crime, making them all eligible for the treatment. However, it is possible that the outcome measures did not accurately assess some of the unchanged individuals’ crimonogenic needs. It is possible that the unchanged participants could be different to the other participants, and that their changing might depend on impacting factors, even though our models could not show this. The unchanged individuals varied between different outcome measures, which highlights the heterogeneity of the offender population. More research is needed into individual offenders’ various backgrounds and needs and treatment impact.

Four participants demonstrated some deterioration between pre-treatment and post-treatment and/or pre-treatment to follow-up, two of these on AQ-RSV and two on either DERS or STAXI. This is an important finding to highlight, since any potentially counterproductive results of a treatment must be known, especially in such a novel treatment as VRAPT. Although deterioration following treatment must be taken seriously, three of these semi-deteriorating participants also seemed to benefit from VRAPT and demonstrated positive changes on other outcome measures. No participant deteriorated across all outcome measures or crossed the cutoff C in the wrong direction. The fourth participant lacked data from post-treatment but deteriorated on all outcome measures except anger expression at follow-up. Given the small sample size, no firm conclusions can be drawn from this, but the findings highlight the importance of matching the treatment to offenders’ individual risks and needs. An important area that this study could not address is how to identify offenders who potentially should not take part in VRAPT and to learn more about the potential hazards and unbeneficial aspects of VR-assisted treatment for offenders. While no in-depth analyses of this is possible with our study data, it is notable that the participants with partial deterioration all scored in the moderate to severe category for emotional neglect and in the severe category for physical neglect according to the CTQ-SF. This could mean that offenders with a history of childhood trauma including physical or emotional neglect might need to be treated differently—or not at all—in VRAPT. Clearly, this needs to be further investigated in future studies as also recommended by Klein Tuente et al. (37).

To further translate the findings from our outcome measures into clinical meaning, they should be placed in a larger context. We found that the sample mean on emotion regulation was below that of a normal population (48) at both post-treatment and follow-up. This was also true for the post-treatment measure of state anger compared with another normal population (50). STAXI in the study sample ranged from the 35 to the 45th percentile for state anger, between the 55 and 70th percentiles for trait anger, and the 55 and 75th percentiles for anger expression at post-treatment and follow-up, putting the sample within a normal population range (50). The study design does not permit conclusions about the effect of the intervention due to the lack of control group, and the findings in this study must be interpreted with much care due to the obvious limitations in the study design and small sample size, but the participants seemed to change on aggression, anger, and emotion dysregulation both during VRAPT and over time.

Several factors may impact the results of this study. For instance, half the sample had participated in treatment programs in the SPPS before entering VRAPT, which could have led to their greater ability to change during the time period for VRAPT due to skills acquired in previous treatments. No analyses of previous treatment in relation to VRAPT outcome were possible in this study, but this highlights a potential way to utilize VR-assisted offender treatment as a means of boosting or rehearsing specific skills and behaviors. Another aspect which must be considered relates to the generalizability of the current results. The current sample—14 violent offenders with high to medium risk of criminal recidivism—was like other offender samples in terms of complexity of needs and background factors commonly found in violent offender populations. Our sample consisted of young individuals lacking in post high school education and work experience, who had a history of externalizing behaviors before age 15. Psychiatric problems and a history of adverse childhood experiences including prior convictions were prominent, as was a history of substance or alcohol abuse. Pro-criminal cognitions and externalizing behaviors over the life span were common. Aggression at baseline as measured by AQ-RSV (M = 94.4, SD = 23.4) was similar to other forensic populations (M = 95.5, SD = 20.4) (86) but differed from the general population (M = 77.8, SD = 16.5) (44). Emotion regulation as measured by DERS (M = 92.9, SD = 23.5 at baseline) was comparable to adults with emotional disorders (M = 89.3, SD = 22.6) (49) but higher than in a general population (M = 78, SD = 20.7) (48). State anger (M = 24, SD = 11.9), trait anger (M = 24.7, SD = 8.1), and anger expression (M = 53.9, SD = 17.9) as measured by STAXI-2-S showed similar levels for state anger (M = 21.3, SD = 1.2) but not trait anger (M = 19.2, SD = 0.84) as a forensic outpatient group (51) but higher than a general population (state anger M = 19.3, SD = 6.9; trait anger M = 18.4, SD = 5.4; anger expression M = 33.5, SD = 13.1) (50). In a general population age group of 20–29, scores between the 25 and the 75th percentiles can be seen as normal (50); the study sample, however, scored in the 80th percentile for state anger, in the 85th percentile for trait anger, and in the 95 to 97th percentiles for anger expression at pre-treatment. The study sample therefore differed from normal populations and demonstrated similarities to clinical samples on the outcome measures. This sample cannot, however, be claimed as representative of violent offenders in general.

Since VR technology is an innovative and novel tool in offender treatment, its impact on offenders is not yet clear. Ethical considerations are important when using persuasive technology such as VR with vulnerable persons (87), and the participants in this study constitute a vulnerable group considering their complex needs, trauma history, and residence under the power dynamics of a correctional facility. The ethical dilemma is how to take vulnerability into consideration, avoid harm, and resist using persuasive technology coercively or manipulatively (87) while still developing better treatment for offenders to reduce their risk of criminal recidivism. A recent systematic review (29), concluded that VR-assisted assessment and treatment does not seem to pose any harm when applied in forensic settings, although much is still unknown of its effects. Thus, utilizing VR within offender treatment is feasible, but we still need to be mindful of its potential impacts and consequences.

Another ethical dilemma involves the risk of not providing potentially beneficial treatment simply because of its unknown characteristics. Standing on the frontlines of treatment development is always foggy, and steps forward need to be taken one at a time before we pick up speed. This study is a small step forward in a small group in dire need of effective treatment. The ethical dilemmas were managed through informed consent and participants’ ability to opt-out (or simply return to the real world by removing the VR goggles) at any time, which were meant to strengthen participant autonomy. All participants received VRAPT as voluntary treatment and were informed that dropping out would not impact their sentence planning or earned prison privileges in any way. The first VRAPT session allows the participant to try the VR environment without any specific purpose except to get acquainted with it. This slow start is important, not only to build trust in the technology, but also to learn more about participants’ reactions. It might be better to let participants try out the VR environment before enrolling in treatment, so they can make a fully informed decision about participating. Another benefit of such an approach would be learning beforehand about participants’ reactions, thoughts, and feelings about VR. This knowledge could be helpful in tailoring the experience better to participants having trouble experiencing immersion and presence in the virtual world and mitigating participants’ fears, attitudes, or prejudice about using VR in treatment.

### 4.1. Strengths and limitations

This study is rare in that it targets imprisoned violent offenders with VR-assisted relapse-preventive treatment, a novel methodology in forensic contexts. The study provides important insights into target group impact and feasibility in an innovative field that has great potential to improve the effectiveness of offender treatment. Specifically, the study provides clinical data on individual change, important knowledge that could be helpful in designing future VR-assisted treatment protocols and studies. The main strengths of the study lie in its novelty and exploratory aspect.

Given the current study’s design as a pilot study, the sample size was small and there was no control group, limiting possible analyses, and conclusions from analyses. When introducing a new treatment protocol based on a novel technology such as VR, pilot studies performed in smaller samples are recommended to limit the number of participants exposed to the intervention in recognition of the importance of testing with care (84). The current analyses were selected considering this, and the combination of both a nomothetic and an idiographic approach with descriptive and statistical analysis filled the explorative purpose. For instance, the small sample made it hard to answer the second research question with inferential statistics on the group level, but the descriptive statistical analysis and analysis of RC made it possible to discuss potential impact factors in line with the aim of the study. To help address the limitation concerning sample size, we report missing post-treatment and follow-up data for different participants. Although this method of linear mixed models can accommodate missing data (88), the fact remains that some analyses consisted of only 11 of 12 participants, which is unfortunate and must be considered when interpreting the results.

The hybrid Bayesian and frequentist approach might also be seen as a limitation in that a more consistent approach would have been preferable. However, from an explorative perspective, the combination of Bayesian linear mixed effects models that could handle the nomothetic approach of group data with a small sample, and the well-established and useful frequentist approach of RCI (89) for the idiographic approach allowed us to utilize the strengths of both Bayesian and frequentist statistics. As stated by Bayarri and Berger (90), statisticians should use both Bayesian and frequentist ideas, and there are several situations where a combination is highly useful.

Another limitation lies in deviations taken from the recommended VRAPT methodology. Only half of the VRAPT treatments were conducted at the recommended intensity, possibly influencing the study’s reliability. Treatment length was, however, both shorter and longer than the recommendations. The prison context is riddled with obstacles for treatment in general due to logistical issues such as overcrowding, staff turnover, competing planned activities, transportation distances, difficulties finding treatment rooms, or client misconduct leading to ward lockdowns. This study was conducted during the years 2020–2022, with a pandemic paralyzing much of the everyday work with both staff and client infections, staff shortages, and ward lockdowns due to infections. In light of both the usual prison constraints and those of the COVID-19 pandemic, impact on treatment length was unavoidable in this study. However, despite these limitations, given the robust RC for many participants in this study, it seems plausible that VRAPT can be adjusted to be implement at different treatment intensities. The strong emphasis on self-reported data to measure change is another limitation in the study. Misconduct could not be used as a change measure mainly due to large differences in time served between participants before VRAPT. Staff ratings of participant behavior which was used in the randomized controlled trial by Klein Tuente et al. (37) was abandoned early in this project due to the overcrowding strain that staff was under at the time, making staff ratings impossible. More precise data on misconduct and staff ratings of participant behavior would certainly have added value to the exploratory aim of the study, providing valuable information on target group impact.

During the study, we found a skew in the number of VRAPT treatments conducted by the individual VRAPT facilitators; some conducted only one treatment, while one conducted as many as 5. Even though all VRAPT facilitators received basic training and supervision in using VR and the VRAPT protocol, it is reasonable to assume that facilitators performing more VRAPT treatments would excel in using the VR technology. With proficiency in using the technology, it is probably easier to focus on alliance and treatment in general. On the other hand, all facilitators were selected because of their experience in offender treatment, CBT methodology, and working with complex violent offenders. Learning a VR-assisted offender treatment program would probably be a much larger challenge for an unexperienced facilitator. Whether treatment effectiveness differs according to the alliance-building and methodological competencies of individual VRAPT facilitators is beyond the scope of this study. It may, however, impact treatment outcomes and should therefore be investigated in further studies since it raises questions about whether VR technology itself is better than the hand wielding it.

Finally, a possible impacting factor that could not be thoroughly investigated in this study was the impact of presence in VR. Our measure of presence in VR, IPQ, unfortunately provided data that was hard to interpret, with large differences in averages between the different subscales, possibly due in part to both general and internal missing data. Also, the IPQ results did not match the feedback provided from the participants during the study, most of whom seemed to describe both proper immersion and presence. Future studies on VR-assisted treatment need to explore further ways to measure presence and immersion, since these concepts are at the core of the VR experience (30).

### 4.2. Future research

Virtual reality-assisted offender treatment is still in its infancy and VRAPT is only one of many potential VR-assisted offender treatment programs. Much is unknown about how, when, with whom, why, and even whether we should use this technology. Randomized controlled trials are needed to evaluate various treatment protocol’s effect on the risk of criminal recidivism. Continued explorative studies on the impact of VR-assisted offender programs and whether and how VR-assisted treatment might trigger trauma responses in offenders and impact treatment responsivity are also needed. Other aims could include determining who might and might not benefit from VR-assisted treatment and whether VR treatment can be tailored to address responsivity issues related to the technology itself such as presence and immersion. Bridging the gap between the participant and the technology raises yet other research questions, and appropriate amounts of VR exposure should also be investigated to establish the most effective treatment intensity. Finally, research is needed on how VR can fit into existing treatment protocols and how clinicians’ experiences using the technology can affect treatment response in participants.

### 4.3. Conclusion

This pilot study provides an early glimpse of how the VR-assisted aggression treatment VRAPT might impact the target group of incarcerated violent offenders. No conclusions on the effect of VRAPT can be made due to the study’s lack of a comparison group, and results must be interpreted with caution due to its small sample size. However, the results indicated that for many participants in this study, a highly probable change in core criminogenic needs related to the risk of relapse in crime occurred during the time for enrollment in VRAPT, and that change was largely maintained over a 3-month period after treatment ended. Some participants did not seem to benefit much from VRAPT treatment, however, and a minority even deteriorated on the outcome measures. From both an ethical and relapse-prevention perspective, further investigations on identifying appropriate VRAPT target groups remain key avenues for future research. Although VRAPT should be considered an early adaptation in a field that will develop rapidly during the years to come, this pilot demonstrates that taking part in VRAPT may be associated with change on important outcomes for imprisoned violent offenders.

## Data availability statement

The raw data supporting the conclusions of this article will be made available by the authors, without undue reservation.

## Ethics statement

The studies involving humans were approved by The Swedish ethical review authority. The studies were conducted in accordance with the local legislation and institutional requirements. The participants provided their written informed consent to participate in this study.

## Author contributions

DI participated in planning the study, collecting, and analyzing the data, and writing and revising the article. CD participated in the data analysis and in writing and revising the article. PE participated in revising the article, and MW planned the study and revised the article. All authors contributed to the article and approved the submitted version.
